# Selective Proteomic Analysis of Antibiotic-Tolerant Cellular Subpopulations in *Pseudomonas aeruginosa* Biofilms

**DOI:** 10.1128/mBio.01593-17

**Published:** 2017-10-24

**Authors:** Brett M. Babin, Lydia Atangcho, Mark B. van Eldijk, Michael J. Sweredoski, Annie Moradian, Sonja Hess, Tim Tolker-Nielsen, Dianne K. Newman, David A. Tirrell

**Affiliations:** a Division of Chemistry and Chemical Engineering, California Institute of Technology, Pasadena, California, USA; b Department of Chemical Engineering, University of Michigan, Ann Arbor, Michigan, USA; c Proteome Exploration Laboratory, Beckman Institute, California Institute of Technology, Pasadena, California, USA; d Costerton Biofilm Center, Department of Immunology and Microbiology, Faculty of Health Sciences, University of Copenhagen, Copenhagen, Denmark; e Division of Geological and Planetary Sciences, California Institute of Technology, Pasadena, California, USA; f Division of Biology and Biological Engineering, California Institute of Technology, Pasadena, California, USA; g Howard Hughes Medical Institute, California Institute of Technology, Pasadena, California, USA; University of Pittsburgh

**Keywords:** BONCAT, *Pseudomonas aeruginosa*, antibiotic resistance, biofilms, proteomics

## Abstract

Biofilm infections exhibit high tolerance against antibiotic treatment. The study of biofilms is complicated by phenotypic heterogeneity; biofilm subpopulations differ in their metabolic activities and their responses to antibiotics. Here, we describe the use of the bio-orthogonal noncanonical amino acid tagging (BONCAT) method to enable selective proteomic analysis of a *Pseudomonas aeruginosa* biofilm subpopulation. Through controlled expression of a mutant methionyl-tRNA synthetase, we targeted BONCAT labeling to cells in the regions of biofilm microcolonies that showed increased tolerance to antibiotics. We enriched and identified proteins synthesized by cells in these regions. Compared to the entire biofilm proteome, the labeled subpopulation was characterized by a lower abundance of ribosomal proteins and was enriched in proteins of unknown function. We performed a pulse-labeling experiment to determine the dynamic proteomic response of the tolerant subpopulation to supra-MIC treatment with the fluoroquinolone antibiotic ciprofloxacin. The adaptive response included the upregulation of proteins required for sensing and repairing DNA damage and substantial changes in the expression of enzymes involved in central carbon metabolism. We differentiated the immediate proteomic response, characterized by an increase in flagellar motility, from the long-term adaptive strategy, which included the upregulation of purine synthesis. This targeted, selective analysis of a bacterial subpopulation demonstrates how the study of proteome dynamics can enhance our understanding of biofilm heterogeneity and antibiotic tolerance.

## IMPORTANCE

Bacterial growth is frequently characterized by behavioral heterogeneity at the single-cell level. Heterogeneity is especially evident in the physiology of biofilms, in which distinct cellular subpopulations can respond differently to stresses, including subpopulations of pathogenic biofilms that are more tolerant to antibiotics. Global proteomic analysis affords insights into cellular physiology but cannot identify proteins expressed in a particular subpopulation of interest. Here, we report a chemical biology method to selectively label, enrich, and identify proteins expressed by cells within distinct regions of biofilm microcolonies. We used this approach to study changes in protein synthesis by the subpopulation of antibiotic-tolerant cells throughout a course of treatment. We found substantial differences between the initial response and the long-term adaptive strategy that biofilm cells use to cope with antibiotic stress. The method we describe is readily applicable to investigations of bacterial heterogeneity in diverse contexts.

## INTRODUCTION

Compared to their planktonic counterparts, bacteria living in surface-associated biofilms are more tolerant to a variety of stresses (1). Of clinical importance is the increased antibiotic tolerance of biofilms, which impedes elimination of chronic infections. Phenotypic tolerance is distinct from genotypic resistance, though the evolution of resistance can be promoted by the persistence of cells following treatment (2). The opportunistic pathogen *Pseudomonas aeruginosa* serves as a model organism for study of both biofilm physiology and antibiotic-tolerant infections. *P. aeruginosa* is a primary contributor to chronic infections of the cystic fibrosis lung, where it forms biofilms that are recalcitrant to the host immune system and antimicrobial therapies. Antibiotic tolerance by these biofilm infections has been documented within the host (3) and through *in vitro* biofilm studies (4).

Research on *P. aeruginosa* biofilms grown *in vitro* has revealed the importance of spatial heterogeneity in their response to antibiotics; specific subpopulations survive treatment while others do not (1, 5). For example, drug classes such as fluoroquinolones (6), aminoglycosides (7), and β-lactams (8), which target active processes (DNA replication, translation, and peptidoglycan synthesis, respectively) kill growing cells within the biofilm regions that have greater access to exogenous nutrients. Explanations for the spatial segregation of these antibiotic responses include reduced penetration of small-molecule antibiotics, decreased metabolic rates, and altered metabolism (1, 5). Conversely, polymyxins and detergents, which disrupt cellular membranes, preferentially kill dormant cells in the interiors of biofilm microstructures (6).

Measurements of mRNA or protein abundance can offer comprehensive and unbiased views of a physiological response to antibiotics (9–11), but experimental challenges limit the investigation of tolerant biofilm subpopulations. Because only a subset of cells exhibit tolerance, any analysis must distinguish tolerant cells from those that do not survive treatment. Laser capture microdissection has been used to isolate biofilm cells from spatially distinct regions of biofilms, and quantitative PCR (qPCR) and DNA microarray analyses have been used to quantify differences in mRNA transcript abundances between these regions (12, 13). This approach has been applied to the exploration of biofilm heterogeneity in general, but not to the study of subpopulation-specific responses to antibiotics. Global proteomic measurements have been widely used to better understand biofilm physiology (14), but targeted selective approaches have been limited.

An important recent advance has been the application of pulsed stable isotope labeling with amino acids in cell culture (pSILAC) to quantify changes in protein expression following adaptation of biofilm cells to challenge with antibiotics (15). Via pulsed addition of an amino acid isotopolog, pSILAC can provide a means to distinguish, based on mass, proteins synthesized before and during the pulse (16). Chua et al. characterized the long-term proteomic response of tolerant cells by treating biofilms with the clinical polymyxin colistin for 8 h to allow nontolerant cells to die and then labeling newly synthesized proteins with an extended (48-h) amino acid isotopolog pulse. This approach ensured that labeled and identified proteins were synthesized over the 2-day period by the tolerant subpopulation of interest. The results of this study revealed the importance of type IV-mediated motility in the resistance of *P. aeruginosa* biofilms to colistin.

To address the challenges posed by dynamic and heterogeneous responses, we employed the bio-orthogonal noncanonical amino acid tagging (BONCAT) method for selective proteomic analysis (17, 18). BONCAT relies on the incorporation into cellular proteins of a noncanonical amino acid (ncAA) that bears a bio-orthogonal chemical handle. Following incorporation, labeled proteins can be conjugated to an affinity tag and enriched from the pool of unlabeled proteins. Enriched proteins can be identified and quantified via liquid chromatography-tandem mass spectrometry (LC-MS/MS). Like pSILAC, BONCAT provides temporal selectivity; proteins synthesized during the ncAA pulse are chemically distinct from preexisting proteins. However, a key advantage of this enrichment-based method is that labeled proteins can be physically separated from the rest of the proteome. MS-based protein identification is sensitive to the complexity of the sample, such that proteins of low abundance often go unidentified; therefore, reducing sample complexity can aid in the detection of proteins of interest. We and others have shown the exquisite temporal sensitivity of BONCAT-based enrichment in the context of dynamic proteome changes (19, 20). In bacteria, ncAA pulse times as short as a few minutes have been used to quantify dynamic processes in *Vibrio harveyi* (21, 22), *Escherichia coli* (23), and *Bacillus subtilis* (24), while extended pulse times have been used to identify proteins synthesized at extremely low rates under conditions of anaerobic survival in *P. aeruginosa* (25).

BONCAT labeling can target cellular subpopulations through the use of an ncAA that is not incorporated into proteins by the endogenous translational machinery. Cells expressing a mutant aminoacyl-tRNA synthetase (mRS) that has been engineered to charge the ncAA to a cognate tRNA are labeled. Such noncanonical synthetases have been developed for the methionine surrogates azidonorleucine (26) and 2-aminooctynoic acid (27) and for the phenylalanine surrogate azidophenylalanine (28). By restricting expression of the mRS to the cell type of interest, protein labeling can focus on a subpopulation of cells within a complex heterogeneous system. Cell targeting can be accomplished by genetically restricting the mRS gene to a species of interest (e.g., bacteria in the presence of host cells [27, 29]) or by placing mRS expression under control of a cell-state-specific promoter (e.g., under reactive oxygen stress conditions for *E. coli* [30]).

Here, we describe an adaptation of the BONCAT method for cell- and time-resolved analysis of protein synthesis in heterogeneous bacterial biofilms. We direct cell-selective labeling of proteins with the ncAA azidonorleucine (Anl) (see Fig. S1 in the supplemental material) through controlled expression of a mutant form of the *E. coli* methionyl-tRNA synthetase (designated NLL-MetRS [26]). By controlling expression of NLL-MetRS with the promoter element for the stationary-phase sigma factor *rpoS*, we restricted protein labeling to the inner *P. aeruginosa* biofilm subpopulation. We used this approach to selectively analyze the dynamic proteomic response of biofilm cells that are tolerant to ciprofloxacin.

10.1128/mBio.01593-17.1 FIG S1  Compounds for BONCAT labeling and enrichment. Download FIG S1, PDF file, 1.6 MB. Copyright © 2017 Babin et al. 2017 Babin et al. This content is distributed under the terms of the Creative Commons Attribution 4.0 International license.

(Parts of this work were conducted as B. M. Babin’s thesis project and are presented in chapter 4 of his dissertation [31].)

## RESULTS

### The *rpoS* promoter enables cell-state-selective labeling.

To selectively target antibiotic-tolerant biofilm cells, we aimed to restrict BONCAT labeling by placing NLL-MetRS expression under control of an endogenous, cell-state-selective promoter. Because biofilm regions more tolerant to many antibiotics contain cells with decreased metabolic rates, we reasoned that the use of a promoter whose activity increases during planktonic stationary phase, when metabolic rates are similarly decreased, would provide the desired selectivity. Cellular levels of the alternative sigma factor σ^38^ are upregulated in response to a variety of stresses. In *P. aeruginosa* and other bacteria, σ^38^, which is encoded by the gene *rpoS*, is upregulated during the transition from exponential to stationary phase during planktonic growth (32); we hypothesized that the *rpoS* promoter would enable selective protein labeling.

**FIG 1:**
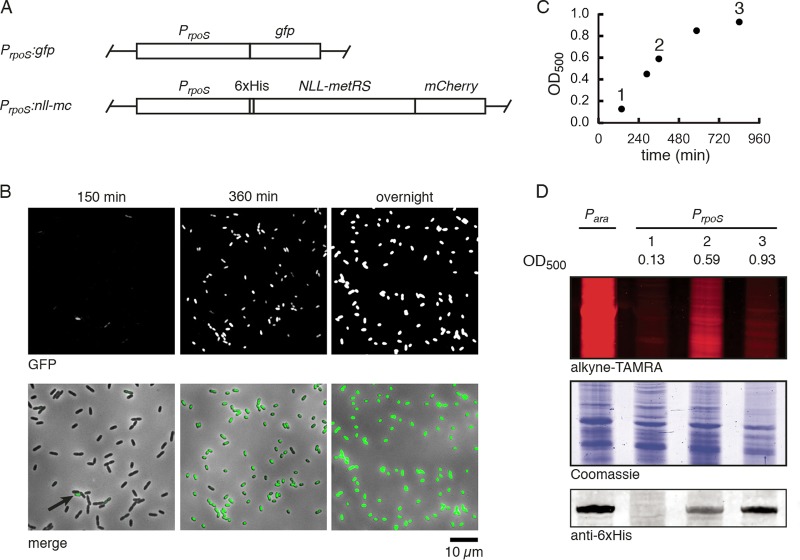
Cell-state-selective labeling using the *rpoS* promoter. (A) *P. aeruginosa* was engineered to express GFP or an NLL-MetRS–mCherry translational fusion under control of the endogenous *rpoS* promoter. Expression cassettes were transposed to the Tn*7* chromosomal locus. (B) Representative images of GFP fluorescence of the *P*_*rpoS*_:*gfp* strain throughout growth in LB medium. GFP fluorescence (top) and a GFP–bright-field merge (bottom) are shown. The arrow indicates a GFP-positive cell at the early time point. The times after 1:200 dilution into fresh medium are indicated above the panels. (C) Optical density at 500 nm of *P*_*rpoS*_:*nll-mc* cells grown in liquid culture in minimal FAB medium. At each indicated time point, an aliquot was removed and incubated with 1 mM Anl for 15 min. (D) Lysates were treated with alkyne-TAMRA and separated via SDS-PAGE to visualize Anl incorporation. Coomassie staining of the same gel indicates equal protein loading. Lysates were probed by Western blotting for the six-histidine tag on NLL-MetRS.

We first evaluated the activity of the *rpoS* promoter in planktonic cells. We placed the 1-kb region upstream of the endogenous *rpoS* gene 5′ to *gfp* and transposed this expression cassette to the Tn*7* locus in *P. aeruginosa* PA14 to generate *P*_*rpoS*_:*gfp* (Fig. 1A). Fluorescence imaging of *P*_*rpoS*_:*gfp* throughout growth from early exponential phase (150 min following dilution) to late stationary phase (overnight) in LB medium revealed the expected increase of promoter activity in high-cell-density, nutrient-depleted cultures (Fig. 1B). We observed cellular heterogeneity in the expression of green fluorescent protein (GFP). At the early time point, only a small subpopulation of cells was GFP positive. The GFP-positive fraction increased in early stationary phase, and after overnight growth essentially all cells expressed GFP. In contrast, wild-type cells exhibited no fluorescence (Fig. S2A), and when GFP expression was placed under control of the strong, constitutive *trc* promoter (33), all cells were GFP positive at all time points (Fig. S2B).

10.1128/mBio.01593-17.2 FIG S2  Promoter-controlled expression. (A and B) Fluorescence images of wild-type (A) and *P*_*trc*_:*gfp* cells grown planktonically. GFP fluorescence (top) and GFP–bright-field merge (bottom) images are shown. (C) Controlled proteome labeling with inducible expression of NLL-MetRS from the *ara* promoter. The SDS-PAGE gel was imaged for TAMRA fluorescence (top) and stained with Coomassie (bottom) to indicate total protein loading. Arrows indicate the NLL-MetRS protein. Download FIG S2, PDF file, 1.9 MB. Copyright © 2017 Babin et al. 2017 Babin et al. This content is distributed under the terms of the Creative Commons Attribution 4.0 International license.

Encouraged by these results, we generated a strain in which expression of NLL-MetRS was controlled by the *rpoS* promoter, again transposed to the Tn*7* locus. The protein was outfitted with an N-terminal six-histidine tag to allow for Western blotting detection and a C-terminal translational fusion to mCherry, yielding *P*_*rpoS*_:*nll-mc* (Fig. 1A). We first verified that this strain allows for Anl labeling under planktonic growth conditions. We grew this strain from early exponential phase to stationary phase in FAB minimal medium and treated samples of the culture with Anl for 15 min at three points throughout growth (Fig. 1C). To provide a positive control, we transformed strain PA14 with a plasmid that enables expression of NLL-MetRS under control of the *P*_*ara*_ arabinose-inducible promoter and treated the transformed cells with arabinose and Anl during exponential phase (Fig. S2C). Consistent with our GFP measurements, Western blotting showed growth phase-dependent expression of NLL-MetRS under control of the *rpoS* promoter (Fig. 1D). To detect Anl incorporation, we took advantage of the selective azide-alkyne cycloaddition (the “click” reaction). We treated cell lysates with alkyne-carboxytetramethylrhodamine (alkyne-TAMRA) (Fig. S1) under copper-catalyzed click conditions, separated proteins via SDS-PAGE, and imaged fluorescence. In early exponential phase, when NLL-MetRS was not present, labeling was not detected. Labeling was strongest in early stationary phase, when NLL-MetRS expression was moderate, and low but detectable in stationary phase. For all conditions, Coomassie staining was used to verify equal protein loading. The rate of Anl incorporation was dependent on both the abundance of NLL-MetRS and the overall rate of protein synthesis during the 15-min Anl pulse, so we interpreted the lower levels of Anl labeling in stationary phase as a reflection of the decreased rates of protein synthesis in this state.

### Labeling and proteomic analysis of a biofilm subpopulation.

**FIG 2:**
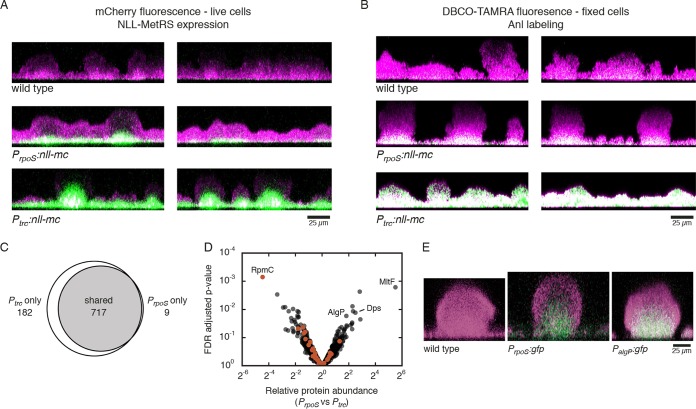
Targeted proteomic analysis of a biofilm subpopulation. (A) Detection of mCherry fluorescence (green) in live biofilms was used to locate cells expressing the NLL-MetRS–mCherry fusion. Biofilms were counterstained with SYTO9 (magenta) immediately before imaging. (B) Following Anl treatment, BONCAT labeling in biofilms was visualized by treating fixed biofilms with DBCO-TAMRA (green). Biofilms were counterstained with SYTO9 (magenta). Colocalization of fluorescent signals is displayed in white. For panels A and B, cross-sections were reconstructed from confocal image stacks. (C) Proteins identified following BONCAT enrichment from *P*_*rpoS*_:*nll-mc* and *P*_*trc*_:*nll-mc* strains. (D) Quantification of relative protein abundances following enrichment from both strains. Ribosomal proteins are shown in orange. Proteins discussed in the text are indicated by gene name. The complete set of LFQ values, ratios, and adjusted *P* values is provided in Data Set S1. (E) Spatial distribution of GFP expression (green) under control of the *rpoS* or *algP* promoters in live biofilms. Biofilms were counterstained with SYTO62 (magenta).

To test for subpopulation targeting in biofilms, we cultured biofilms on glass surfaces under constant medium flow in flow cells. Four-day-old live biofilms were analyzed via confocal microscopy. SYTO 9 was used as a cell stain, and mCherry fluorescence was used to locate cells expressing the NLL-MetRS–mCherry fusion (Fig. 2A). *P*_*rpoS*_:*nll-mc* exhibited mCherry fluorescence only in the lower parts of biofilm structures, while a strain expressing NLL-MetRS from a strong constitutive promoter (*P*_*trc*_:*nll-mc*) exhibited mCherry fluorescence throughout the biofilm structures. Because only cells that express NLL-MetRS can incorporate Anl, we expected that the difference in expression patterns would allow for subpopulation-specific BONCAT labeling.

To visualize BONCAT labeling, we again cultured biofilms in flow cells for 4 days and then treated them with Anl for 1.5 h. Anl incorporation was visualized by treating fixed biofilms with aza-dibenzocyclooctyne (DBCO)-TAMRA (Fig. S1). The strained alkyne structure present in DBCO allows for copper-free azide-alkyne cycloaddition and removes the requirement for diffusion of the ligand, copper catalyst, and reductant needed for conjugation of alkyne-TAMRA. Cells were counterstained with SYTO 9 (Fig. 2B). As expected, we observed similar patterns in Anl labeling as for NLL-MetRS expression. *P*_*rpoS*_:*nll-mc* incorporated Anl predominantly in the lower parts of biofilm structures, and *P*_*trc*_:*nll-mc* incorporated Anl throughout the entirety of the biofilm. Wild-type cells exhibited minimal background fluorescence, likely due to nonspecific interactions between DBCO-TAMRA and the cells.

To evaluate our ability to detect proteins preferentially expressed by the labeled subpopulation, we compared proteomes enriched from *P*_*trc*_:*nll-mc* and *P*_*rpoS*_:*nll-mc* strains. To obtain adequate protein yields, biofilms of each strain were grown for 4 days in silicone rubber tubing and treated with Anl for 1.5 h before cells were collected and lysed. We verified Anl incorporation by treating cell lysates with alkyne-TAMRA and analyzing TAMRA fluorescence via SDS-PAGE (Fig. S3A). We observed a statistically significant increase in fluorescence for *P*_*trc*_*:nll-mc* and *P*_*rpoS*_:*nll-mc* samples compared to a methionine-treated control (*P* < 0.01 for all) (Fig. S3B). To enable enrichment of Anl-labeled proteins, we treated lysates with DBCO–sulfo-biotin (Fig. S1). Western blot detection using streptavidin revealed substantial biotinylation of Anl-labeled proteins and three bands in the methionine-treated control, likely corresponding to endogenously biotinylated proteins (Fig. S3C). Three of four Anl-treated samples showed a significant increase in biotinylation (*P* < 0.05) (Fig. S3D).

10.1128/mBio.01593-17.3 FIG S3  Enrichment from biofilms of *P*_*rpoS*_:*nll-mc* and *P*_*trc*_:*nll-mc* cells. (A) SDS-PAGE analysis showing Anl incorporation for each biofilm replicate. Coomassie indicates protein loading. Biofilms were treated with 1 mM Anl or 1 mM methionine (Met) where indicated. (B) Anl incorporation was quantified by dividing TAMRA fluorescence by Coomassie intensity for four gel regions (means + standard regions; *n* = 4), **, *P* < 0.01 (Welch’s *t* test). (C) Western blot with streptavidin-Alexa Fluor 488 conjugates of lysates treated with DBCO–sulfo-biotin for affinity enrichment. Three naturally biotinylated proteins are visible in the methionine-treated negative control. (D) Reaction of Anl-modified proteins with the biotin probe was quantified by dividing Alexa Fluor 488 fluorescence by Coomassie intensity for four regions (means + standard deviations; *n* = 4). *, *P* < 0.05 (Welch’s *t* test). (E) Distribution of protein abundance ratios for all proteins (gray) and for ribosomal proteins (orange). Each box indicates the second and third quartiles, and whiskers indicate the rest of the distribution. Values exceeding 1.5 times the interquartile range are indicated as points. The *P* values were calculated by bootstrapped subsampling from the set of all abundance ratios (subsampled *n* = 39 ratios, bootstrapped 1,000 times). Download FIG S3, PDF file, 2.8 MB. Copyright © 2017 Babin et al. 2017 Babin et al. This content is distributed under the terms of the Creative Commons Attribution 4.0 International license.

Anl-labeled proteins were enriched on streptavidin beads and analyzed via LC-MS/MS. We detected 908 proteins among two replicates from each strain, with fewer identifications from the *P*_*rpoS*_:*nll-mc* strain (726 [80%]) (Fig. 2C). Based on in-gel fluorescence and Western blot detection (Fig. S3A to D), Anl incorporation was not lower in these samples, so the decreased number of identified proteins from the *P*_*rpoS*_:*nll-mc* samples was likely due to the targeted analysis of a subset of biofilm cells. We quantified relative abundances of proteins identified from both strains via label-free quantification (LFQ) and found the abundance levels of 15 and 24 proteins to be at least 2-fold increased or decreased in the *P*_*rpoS*_:*nll-mc* strain, respectively (Fig. 2D; Tables S1 and S2). Full proteomic results from this experiment are provided in Data Set S1.

10.1128/mBio.01593-17.5 TABLE S1  Proteins significantly more abundant in the *P*_*rpoS*_ biofilm region. Download TABLE S1, XLSX file, 0.03 MB. Copyright © 2017 Babin et al. 2017 Babin et al. This content is distributed under the terms of the Creative Commons Attribution 4.0 International license.

10.1128/mBio.01593-17.6 TABLE S2  Proteins significantly less abundant in the *P*_*rpoS*_ biofilm region. Download TABLE S2, XLSX file, 0.04 MB. Copyright © 2017 Babin et al. 2017 Babin et al. This content is distributed under the terms of the Creative Commons Attribution 4.0 International license.

10.1128/mBio.01593-17.8 DATA SET S1  Proteomic results for BONCAT analysis of *P*_*trc*_:*nll-mc* and *P*_*rpoS*_:*nll-mc* biofilms. All proteins identified by LC-MS/MS from the BONCAT-enriched samples are listed. Proteins are identified by Uniprot ID, NCBI GI Number, RefSeq Accession, PA14 locus ID, PAO1 homolog locus ID (when available), Gene Name, Protein Description, and PseudoCAP annotations. Protein abundances for each MS sample are reported as calculated by MaxQuant (LFQ). A value of 0 indicates that a protein was not identified in that replicate. Protein ratios of median abudances are given as the log_2_(*P*_*rpoS*_ LFQ/*P*_*trc*_ LFQ ratio), with corresponding adjusted *P* values. Download DATA SET S1, XLSX file, 0.2 MB. Copyright © 2017 Babin et al. 2017 Babin et al. This content is distributed under the terms of the Creative Commons Attribution 4.0 International license.

We hypothesized that the subpopulation labeled in the *P*_*rpoS*_:*nll-mc* strain would exhibit decreased metabolic rates. We looked for differences in ribosome synthesis and found that ribosomal proteins were significantly less abundant in samples enriched from the *P*_*rpoS*_:*nll-mc* strain (*P* < 0.05). The median relative abundance of 36 quantified ribosomal proteins was 1.7-fold lower in *P*_*rpoS*_:*nll-mc* samples than in the *P*_*trc*_:*nll-mc* samples (Fig. S3E). Furthermore, the protein with the lowest relative abundance in the *P*_*rpoS*_:*nll-mc* samples was the nonessential ribosomal protein RpmC (protein L29 of the 60S subunit; 22-fold less abundant). These results are consistent with measurements showing greater translational activity in the upper regions of flow cell biofilms (34) and the higher levels of transcripts for ribosomal components found in highly metabolically active versus less-active cells within colony biofilms (13).

Proteins with known functions found to be enriched in the *P*_*rpoS*_:*nll-mc* samples included those involved in antibiotic resistance, stress protection, and alginate regulation (Fig. 2D). The lytic transglycosylase MltF (accession number PA14_15720), was the protein most enriched in the *P*_*rpoS*_:*nll-mc* subpopulation. MltF and other transglycosylases involved in peptidoglycan remodeling have been implicated in resistance to the β-lactams piperacillin, cefotaxime, and ceftazidime, though there are conflicting reports on the effects of *mltF* disruption on MICs (35,36). Biofilm resistance to β-lactams has been linked previously to upregulation of the β-lactamase AmpC in peripheral cells in response to antibiotic treatment (8). The identification of MltF upregulation in the absence of antibiotic stimulation and within the biofilm interior supports a complementary approach to tolerance in which cells are preemptively prepared for antibiotic stress.

We found the starvation factor Dps to be significantly more abundant in the *P*_*rpoS*_:*nll-mc* enrichment experiment. Dps is a global DNA remodeling protein that confers protection against a variety of stresses, including nutrient limitation, oxidative stress, UV irradiation, and others (37,38). In *E. coli*, *dps* transcription is RpoS dependent, and Dps is among the most abundant proteins in stationary-phase cells (39). The higher abundance of Dps in the biofilm interior is consistent with the expected nutrient deprivation of cells in this region.

We also observed the DNA binding protein AlgP to be significantly more abundant in the *P*_*rpoS*_:*nll-mc* enrichment. AlgP is a regulator reported to be involved in the mucoid phenotype and in the synthesis of the exopolysaccharide alginate (40), though its regulatory role may be more general. AlgP contains a histone-like domain, and immunostaining combined with electron microscopy has revealed association of AlgP with nucleoid fibril structures (41), a finding reminiscent of other histone-like bacterial proteins that play roles in nucleoid architecture and global regulation (42). Because AlgP has not been reported to be specifically expressed in biofilms or in slow-growing cells, we chose this protein to validate the ability of our targeted proteomics approach to provide information about region-specific protein expression. We grew biofilms from a strain that expresses GFP under control of the *algP* promoter (*P*_*algP*_:*gfp*). After 4 days of growth, GFP fluorescence in *P*_*algP*_:*gfp* biofilms was visualized via confocal microscopy. As predicted by our proteomic measurements, expression from the *algP* promoter was restricted to cells within the biofilm interior (Fig. 2E). This pattern of expression matched the localization of GFP fluorescence in *P*_*rpoS*_:*gfp* biofilms (Fig. 2E) and the localization of NLL-MetRS–mCherry expression (Fig. 2A) and Anl labeling (Fig. 2B) observed in *P*_*rpoS*_:*nll-mc* biofilms.

As a caveat, we note that the RpoS protein itself was equally abundant in the *P*_*rpoS*_:*nll-mc* and *P*_*trc*_:*nll-mc* samples. The design of the expression cassette in *P*_*rpoS*_:*nll-mc* places NLL-MetRS under transcriptional control of any regulatory regions that lie 1 kb upstream of the endogenous *rpoS* gene. However, much of the control of RpoS protein levels is known to be posttranscriptional, depending on the action of sRNAs, modified translation rates, and tuned degradation (43). Additionally, NLL-MetRS was fused to mCherry, which may increase its intracellular stability and further disconnect levels of the mutant synthetase from levels of RpoS itself. We conclude that *P*_*rpoS*_:*nll-mc* cells with high NLL-MetRS abundance are not necessarily cells with high RpoS abundance. However, our imaging results from planktonic and biofilm growth states showed that the *P*_*rpoS*_:*nll-mc* strain can be used to target proteomic analysis to the cellular subpopulation of interest.

### BONCAT enrichment of proteins synthesized during ciprofloxacin treatment.

**FIG 3:**
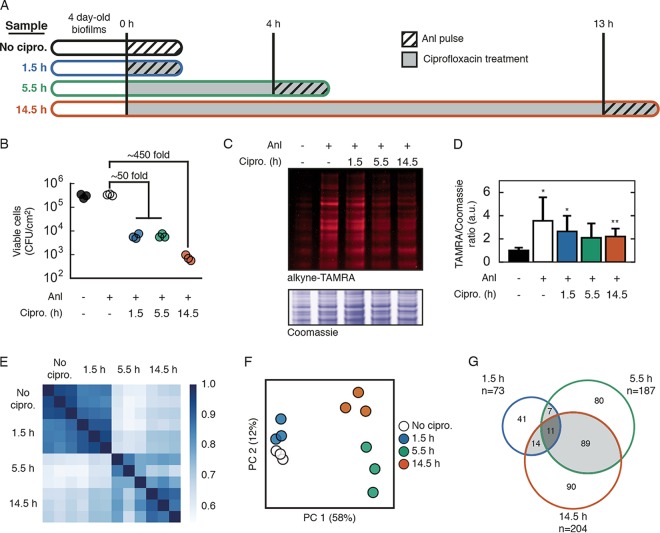
BONCAT analysis of protein synthesis during ciprofloxacin treatment. (A) Experimental timeline of biofilm treatment and proteome labeling. Biofilms were grown in silicone rubber tubing for 4 days and then treated with 60 μg/ml ciprofloxacin (gray). Control biofilms were untreated. For each condition, biofilms were treated with Anl at the designated time point for 1.5 h (cross-hatched portion), harvested, and lysed. (B) Survival of biofilm cells following exposure to ciprofloxacin for the indicated treatment time and to 1 mM Anl as indicated. (C) Visualization of Anl incorporation in lysates treated with alkyne-TAMRA. Coomassie staining was used to verify equal protein loading. (D) Anl incorporation was quantified by dividing the TAMRA fluorescence by the Coomassie intensity for four gel regions (means + standard deviations; *n* = 4). Welch’s t-test results are indicated: *, *P* < 0.05; **, *P* < 0.01. (E) Spearman rank correlation coefficients for protein LFQ values, calculated among all MS runs. (F) The top two principal component weights for each MS run. The percent variance explained by each component is shown in parentheses. (G) Overlap of significantly changed or uniquely identified proteins (down- and upregulated proteins) at each time point throughout ciprofloxacin treatment.

To identify the subpopulation-specific response to ciprofloxacin, we designed an experiment to capture dynamic changes in the proteome throughout the course of antibiotic challenge. A previous study using fluorescent imaging of biofilms treated with ciprofloxacin showed a progression of cell death over the course of 13 h (6). Cell death, visualized by propidium iodide staining, began between 4 and 9 h of treatment and was restricted to peripheral regions of biofilm microstructures. Protein synthesis, measured by expression of an unstable GFP variant, continued in interior biofilm populations even after 13 h of treatment. We sought to investigate the set of proteins made by biofilm cells surviving in the presence of ciprofloxacin. We replicated this time course of antibiotic challenge by treating 4-day-old *P*_*rpoS*_:*nll-mc* biofilms with 60 µg/ml ciprofloxacin. To achieve temporal selectivity, we pulse-labeled biofilms with Anl at 0, 4, or 13 h after ciprofloxacin was added. Each pulse was for 1.5 h to distinguish proteins synthesized during this short pulse from the preexisting proteome. To serve as a no-ciprofloxacin control, we also labeled untreated biofilms for 1.5 h with Anl (Fig. 3A).

Given the evidence that the *P*_*rpoS*_:*nll-mc* strain targets labeling to cells in the biofilm regions known to tolerate ciprofloxacin, we expected that the majority of cells incorporating Anl would remain alive throughout treatment. We tracked the number of viable cells recovered from biofilms throughout the time course of treatment and found two stages of killing (Fig. 3B). Compared to untreated biofilms, those treated for 1.5 or 5.5 h both exhibited an approximately 50-fold loss in viable cells, while those treated for 14.5 h exhibited an approximately 450-fold loss. Treatment with Anl had no effect on the number of viable cells in untreated biofilms (*P* = 0.38, Welch’s *t* test). To quantify Anl incorporation in ciprofloxacin-treated biofilms, we treated lysates with alkyne-TAMRA and analyzed fluorescence via SDS-PAGE (Fig. 3C and D). Strikingly, total Anl incorporation decreased less than 2-fold throughout the course of ciprofloxacin treatment. Even after 14.5 h of treatment, when only 0.2% of biofilm cells remained viable, the level of Anl incorporation by surviving cells (as measured by the ratio of TAMRA fluorescence to Coomassie staining in protein gels) was similar to that observed for untreated cells. In this type of experiment, total Anl incorporation depends on the number of cells expressing NLL-MetRS and the relative rates of protein synthesis and protein degradation in these cells during each 1.5-h Anl pulse. We note that in-gel fluorescence measurements of Anl incorporation cannot distinguish these contributing factors. Together, these results implied that while a majority (99.8%) of biofilm cells died over 14.5 h, the number of cells susceptible to Anl labeling remained nearly constant during this period.

We performed BONCAT enrichment on lysates from each experimental condition (performed in triplicate) and identified proteins by using LC-MS/MS. We identified more than 1,200 proteins among all runs. Protein abundances, estimated by LFQ, were well correlated among replicates for each time point (Fig. 3E). We used principal component (PC) analysis to visualize the variance among replicates and experimental conditions (Fig. 3F) and found that biological replicates clustered with one another and that ciprofloxacin-treated samples were separated from the untreated control samples. For each time point, we compared protein LFQ values in the treated samples and the no-ciprofloxacin control, which yielded ratios for each protein that represented its relative abundance (e.g., up- or downregulation) in treated and untreated samples. Consistent with the correlation analysis, fewer proteins were significantly changed under the 1.5-h treatment condition (73 proteins) than under the 5.5-h treatment (187 proteins) or 14.5-h treatment (204 proteins) (Fig. 3G). Correlation, PC, and relative abundance analysis results were consistent with the classification of the proteomic data into two subgroups: (i) a smaller group of proteins whose synthesis changed immediately upon ciprofloxacin exposure, and (ii) a larger group of late response proteins, many of which were shared between the 5.5-h and 14.5-h groups.

For subsequent analyses, we divided proteins identified at each time point into subsets of groups significantly more or less abundant in the treated versus untreated samples. Each group included proteins whose relative abundances were quantified (fold change of >2 and false-discovery rate [FDR] adjusted *P* value of <0.05), as well as proteins identified under one condition (i.e., found in at least two of three replicates) but not identified under the other (i.e., not found in any replicates). Full proteomic results are listed in Data Set S2.

10.1128/mBio.01593-17.9 DATA SET S2  Proteomic results for BONCAT analysis of ciprofloxacin-treated biofilms. All proteins identified by LC-MS/MS from the BONCAT-enriched samples are listed. Proteins are identified by Uniprot ID, NCBI GI Number, RefSeq Accession, PA14 locus ID, PAO1 homolog locus ID (when available), Gene Name, Protein Description, and PseudoCAP annotations. Protein abundances for each MS sample are reported as calculated by MaxQuant (LFQ). A value of 0 indicates that a protein was not identified in that replicate. Protein ratios of median abudances are given as the log_2_(time point versus No Cipro. LFQ ratio), with corresponding adjusted *P* values. Download DATA SET S2, XLSX file, 0.3 MB. Copyright © 2017 Babin et al. 2017 Babin et al. This content is distributed under the terms of the Creative Commons Attribution 4.0 International license.

### The dynamic proteomic response to ciprofloxacin.

**FIG 4:**
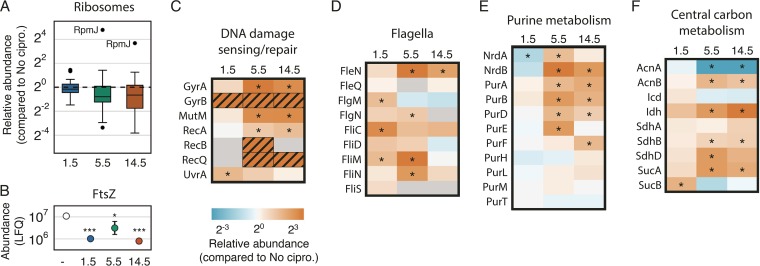
Dynamic cellular responses to ciprofloxacin. (A) Box plots showing the distribution of ribosomal protein abundances for treated samples compared to the untreated control. Each box indicates the second and third quartiles, and whiskers indicate the rest of the distribution. Values exceeding 1.5 times the interquartile range are displayed as points. The nonessential ribosomal protein RpmJ is indicated. (B) Abundance of FtsZ in each sample (mean ± standard deviation; *n* = 3; FDR adjusted *P* values are designated: *, *P* < 0.05; ***, *P* < 0.001). (C to F) Heat maps indicating the median abundance ratio for each time point compared to the untreated control for proteins involved in DNA damage sensing and repair (C), flagellum synthesis (D), purine metabolism (E), and central carbon metabolism (F). *, *P* < 0.05 (FDR-adjusted *P* value). The color scale for abundance ratios is shown under panel C. Gray boxes indicate time points for which that protein was not quantitated. Hatched orange boxes indicate proteins that were identified for that time point (in at least two replicates) but absent from the untreated control. An example of raw abundance measurements for panel C is given in Fig. S4. The complete set of LFQ values, ratios, and adjusted *P* values is provided in Data Set S2.

Based on total Anl incorporation, protein translation rates were reduced in the 5.5- and 14.5-h samples. To test whether this decrease could be explained by changes in ribosome expression, we examined the relative abundance of ribosomal proteins at each time point compared to those for the no-ciprofloxacin control (Fig. 4A). Ribosomal protein abundances were essentially unchanged after 1.5 h of treatment but decreased more than 1.5-fold after 5.5 and 14.5 h of treatment. Interestingly, the nonessential ribosomal protein RpmJ (L36 of the 50S subunit) was substantially upregulated at each of the later time points. We also found a significant decrease in the cell division protein FtsZ under all ciprofloxacin-treated conditions (Fig. 4B). Together, these findings suggest that translation rates and cell division decrease upon ciprofloxacin exposure.

We next compared our results to known responses of *P. aeruginosa* to ciprofloxacin. The responses of planktonic *P. aeruginosa* to sub-MIC and supra-MIC ciprofloxacin treatments (0.01 to 1 μg/ml) have been characterized via microarray and RNA sequencing measurements of transcript abundances (10, 44–46). While the designs of these experiments differed, we found two genes identified in all studies that showed consistent responses to ciprofloxacin. This core response comprises genes involved in the SOS response: LexA and RecA. Ciprofloxacin inhibits DNA gyrase activity, causing DNA damage during replication attempts. DNA damage leads to an induction of the SOS response, characterized by depletion of the SOS repressor LexA and the consequent upregulation of genes that alleviate DNA damage stress (47). We did not identify LexA in any experiments, but we detected upregulation of the LexA target RecA, which binds to DNA lesions. Of the proteins involved in DNA damage response and repair found in our data, all were either significantly upregulated or uniquely identified for at least one time point (Fig. 4C; Fig. S4). This set includes both subunits of DNA gyrase (GyrA and GyrB), the direct target of ciprofloxacin, and proteins involved in sensing (RecA and UvrA) and repairing (RecB, RecQ, and MutM) various types of DNA damage. The identification of these proteomic changes validated the experimental approach and suggests that our results represent *bona fide* physiological responses to ciprofloxacin.

10.1128/mBio.01593-17.4 FIG S4  Changes in protein abundances throughout ciprofloxacin treatment for proteins involved in DNA damage sensing and repair. For all plots, the vertical axis represents protein abundance (LFQ). Individual protein abudances (gray circles) and median abundances (dashes) are indicated for all replicates, for all time points. FDR-adjusted *P* values: *, *P* < 0.05; **, *P* < 0.01; ***, *P* < 0.001. If a protein was not identified for a given time point, no values are plotted. Download FIG S4, PDF file, 1.3 MB. Copyright © 2017 Babin et al. 2017 Babin et al. This content is distributed under the terms of the Creative Commons Attribution 4.0 International license.

As described above, early and late responses to ciprofloxacin differed in our data set. In fact, only three proteins were significantly upregulated at all time points: GyrB, the protein chaperone HscK, and the methylisocitrate lyase PrpB. These proteins typify functional categories of proteins we found to be upregulated by ciprofloxacin challenge, namely, those involved in remediating DNA damage and other stresses and proteins involved in central metabolism. The differences between early and late responses are best exemplified by the contrasting behavior of proteins involved in flagellar synthesis and purine metabolism. Protein components of flagella and flagellar regulation were significantly upregulated at the early and middle time points of ciprofloxacin treatment, including the immediate upregulation of FliC, FliM, and FlgM (Fig. 4D). In contrast, many proteins involved in purine metabolism were upregulated only at the 4.5- and 14.5-h time points (Fig. 4E). Of the proteins in the pathway for *de novo* synthesis of IMP that we identified (PurBDEFHLMT), four of eight were significantly upregulated in at least one of the later time points. PurA, required for conversion of IMP to AMP, was likewise upregulated. The ribonucleotide reductase complex (NrdA and NrdB) that generates deoxyribonucleotides from their ribonucleotide precursors was also upregulated.

Finally, some of the largest changes we observed were the up- or downregulation of proteins involved in central metabolism, particularly components of the citrate (tricarboxylic acid [TCA]) cycle (Fig. 4F). Of particular interest was the differential behavior of the aconitases and isocitrate dehydrogenases (ICDs). The *P. aeruginosa* genome encodes two of each protein class (aconitases AcnA and AcnB and ICDs Icd and Idh). We found AcnA to be significantly downregulated and AcnB to be significantly upregulated in the later stages of treatment. Similarly, expression of Icd, the monomeric ICD, was unchanged, while the dimeric Idh was the protein most upregulated at the 14.5-h time point (40-fold more abundant than in the untreated control).

## DISCUSSION

Here, we introduced an adaptation of BONCAT for selective, time-resolved proteomic analyses of phenotypically distinct subpopulations of cells in genotypically uniform bacterial cultures. As an example, we showed that this method reveals distinct patterns of protein synthesis by antibiotic-tolerant biofilm cells following extended incubation with supra-MIC levels of ciprofloxacin. While the data reported here provide guidance and motivation for future mechanistic studies, the most important result of this work is the illustration of the power of BONCAT to resolve proteome dynamics in space and time in phenotypically heterogeneous microbial systems. This method has the potential to advance a variety of studies in the biofilm field.

Our observations provide evidence for both passive and active tolerance mechanisms in biofilms. A critical factor known to influence bacterial tolerance to antibiotics is metabolic rate; cells with lowered rates of transcription, translation, and cell division can survive inhibition of these processes, which are essential for growing cells (48, 49). Prior to antibiotic exposure, cells in the biofilm interior had lower rates of ribosomal protein synthesis and higher levels of the stationary-phase stress protein Dps, suggesting that the region targeted by the *rpoS* promoter is populated by cells with stationary-phase-like physiology and decreased metabolic rates. Ciprofloxacin exposure led to reduced ribosome synthesis and downregulation of the cell division protein FtsZ, indicating a shift by tolerant cells away from growth. Concurrent with the decrease in ribosome synthesis, proteins important for responding to and repairing DNA damage caused by ciprofloxacin (e.g., the SOS response) were upregulated. This analysis suggested a coordinated shift away from active growth and toward surveillance and repair of the effects of the stresses caused by treatment.

Although ribosome synthesis decreased, translation continued in tolerant cells, even following extended ciprofloxacin treatment. After 14.5 h of antibiotic treatment, total Anl incorporation decreased less than 2-fold compared to the untreated control. Colony counts of viable cells showed a 99.8% decrease in cell numbers by the end of the time course, while our imaging data showed that, in the absence of antibiotic, substantially more than 0.2% of *P*_*rpoS*_:*nll-mc* biofilm cells expressed NLL-MetRS–mCherry and were labeled by Anl. One explanation to resolve this apparent discrepancy is that not all cells that are translationally active within treated biofilms can be cultured following biofilm disruption. Cells that are viable but nonculturable (VBNC) are observed in diverse bacterial systems and can be induced by nutrient starvation and oxygen limitation, conditions characteristic of biofilm interiors (50). For example, planktonic *E. coli* cultures treated with supra-MIC levels of ciprofloxacin have been shown to maintain membrane potential and protein synthetic activity, despite log decreases in culturable cells (51). BONCAT, as applied here, cannot differentiate translation by culturable cells, VBNCs, or dying cells, but we suspect that the number of surviving cells may be larger than that measured by colony counting.

The adaptive response of the *P. aeruginosa* biofilm subpopulation presented here leads to interesting questions about the role of proteome remodeling in ciprofloxacin tolerance. For example, upregulation of flagellar proteins is a hallmark of biofilm dispersal that occurs as biofilms age (52) or in response to nutrient shifts (53). The dispersal response is characterized by upregulation of the flagellar filament FliC, which we found to be upregulated immediately in response to ciprofloxacin challenge. We also found large changes in the abundance of proteins with roles in central carbon metabolism. In this category, we found differential expression of enzymes with similar catalytic functions, i.e., the ICDs Idh (upregulated) and Icd (unchanged) and the aconitases AcnA (downregulated) and AcnB (upregulated). To our knowledge, broad adjustments to central carbon metabolism in *P. aeruginosa* biofilms have not been reported as a mechanism for ciprofloxacin tolerance. A recent study, however, identified the TCA metabolites fumarate and glyoxylate as key modulators of *P. aeruginosa* susceptibility to the aminoglycoside tobramycin (54). Does the early upregulation of flagellar proteins promote mobilization of biofilm cells? How do the changes to the central carbon proteome affect the intracellular redox balance and intermediate metabolite concentrations? What is the physiological benefit of the differential expression of functionally redundant enzymes like the aconitases and ICDs? Dissecting the roles of these proteins and others in the data set through focused genetic experiments and metabolomics will lead to better understanding of these phenomena.

Study of the role of heterogeneity in bacterial physiology is hindered by the difficulties of separating cell types of interest from their phenotypically distinct neighbors. Because cell-selective BONCAT can be targeted using genetic regulatory elements, in principle the method is applicable to any genetically tractable organism. This work describes methods for validating selective NLL-MetRS expression via a fluorescent protein fusion, validating selective Anl incorporation via fixed cell imaging and SDS-PAGE analysis, and for enriching and identifying proteins synthesized by a small subpopulation of targeted cells. The BONCAT approach is readily adaptable to other organisms and we expect these methods will be useful for the analysis of other heterogeneous systems (e.g., planktonic persister cells or *in vivo* infections).

## MATERIALS AND METHODS

### Strain construction.

All strains used are listed in Table S3 and were generated using standard cloning procedures. Enzymes were purchased from New England BioLabs. For chromosomal integration into the Tn*7* site, pUC18T mini-Tn*7*T was modified with the desired expression cassette, followed by tetraparental conjugation to the PA14 host strain as previously described (55). Genomic DNA was prepared using the GenElute bacterial DNA kit (Sigma-Aldrich). The 1-kb regions upstream of *rpoS* and *algP* were amplified from *P. aeruginosa* genomic DNA. GFP-expressing cassettes contained the gene for gfpmut3b cloned from pBK-mini-Tn*7*-gfp2 (56). The gene encoding the *E. coli* mutant methionyl-tRNA synthetase was cloned from plasmid pJTN1 (29). A shuttle vector encoding arabinose-inducible expression of NLL-MetRS was created by cloning the gene from pJTN1 into pBAD18 (57) and then ligating the fragment containing *araC* and *P*_*ara*_:*nll* into pUCP24 (58) to generate pBADP-NLL. *P. aeruginosa* was transformed by electroporation.

10.1128/mBio.01593-17.7 TABLE S3  Bacterial strains and plasmids used in this study. Download TABLE S3, DOCX file, 0.1 MB. Copyright © 2017 Babin et al. 2017 Babin et al. This content is distributed under the terms of the Creative Commons Attribution 4.0 International license.

### Media and growth conditions.

Planktonic cultures were grown at 37°C with shaking. Liquid media were LB (5 g yeast extract, 10 g tryptone, 10 g NaCl per liter), or FAB [2 g (NH_4_)_2_SO_4_, 6 g Na_2_HPO_4_ ⋅ 2H_2_O, 3 g KH_2_PO_4_, 3 g NaCl per liter] with 0.1 mM CaCl_2_, 1 mM MgCl_2_, and 1 ml/liter trace metals mix (200 mg/liter CaSO_4_ ⋅ 2H_2_O, 200 mg/liter FeSO_4_ ⋅ 7H_2_O, 20 mg/liter MnSO_4_ ⋅ H_2_O, 20 mg/liter CuSO_4_ ⋅ 5H_2_O, 20 mg/liter ZnSO_4_ ⋅ 7H_2_O, 10 mg/liter CoSO_4_ ⋅ 7H_2_O, 12 mg/liter NaMoO_4_ ⋅ H_2_O, and 5 mg/liter H_3_BO_3_). Carbon was supplied to FAB by addition of 0.05 g/liter glucose (for biofilms) or 5 g/liter glucose (for planktonic cultures) (59). For confocal imaging, biofilms were grown in flow cells (1 by 4 by 40 mm; Stovall) as previously described (60), but without bubble traps. Biofilms were grown at 37°C with a constant flow rate of 0.03 ml/min. For proteomic analyses, biofilms were grown in silicone rubber tubing (10-mm interior diameter, 20 cm long; McMaster-Carr) at 37°C with a constant flow rate of 0.5 ml/min, as previously described (61). Loosely adherent biofilm cells were extracted by collecting media within each tube and flushing with 0.9% NaCl. Tubing was cut into 1-cm pieces and vortexed in 0.9% NaCl to remove remaining cells. All cells were combined. To count viable cells, cells were washed once with phosphate-buffered saline (PBS) and serial dilutions were made from each sample, spotted onto LB agar, and allowed to grow for 16 h at 37°C.

### BONCAT labeling and enrichment.

For planktonic cell labeling experiments, strains from overnight cultures were diluted 1:100 into FAB medium with 5 g/liter glucose. At each time point, labeling was initiated by the addition of 1 mM Anl (Iris-Biotech). The strain containing pBADP-NLL-MetRS was grown in the presence of 50 μg/ml gentamicin and treated with 1 mM Anl and 20 mM arabinose. For all, after 15 min of incubation with Anl at 37°C with shaking, cells were pelleted at 4°C, washed once with ice-cold 0.9% NaCl, and frozen at −80°C. For biofilm experiments, flow was stopped and tubing was clamped. FAB medium with 0.05 g/liter glucose and 1 mM Anl was injected by syringe, and biofilms were incubated for 1.5 h at 37°C. For ciprofloxacin-treated samples, 60 µg/ml ciprofloxacin was included in the labeling medium. For proteomic analysis, cells were collected from tubing as described above, pelleted, and frozen at −80°C.

All samples were lysed by resuspension in lysis buffer (100 mM Tris-HCl [pH 8], 4% SDS). Lysates were sonicated with a microtip probe for 30 s at a setting of 20% (Qsonica). For Western blot detection of NLL-MetRS, 10 µg of each lysate was separated by SDS-PAGE, transferred to nitrocellulose (GE Healthcare), and probed with penta-His Alexa Fluor 488 (Qiagen). For fluorescence detection of Anl-labeled proteins, lysates were incubated with 5 μM alkyne-TAMRA (Click Chemistry Tools), 100 μM CuSO_4_, 500 μM Tris(3-hydroxypropyltriazolylmethyl)amine (THPTA; Click Chemistry Tools), 5 mM aminoguanidine hydrochloride, and 5 mM sodium ascorbate for 15 min at room temperature (62). The mixtures were then precipitated with water, methanol, and chloroform and washed twice with methanol. TAMRA-labeled lysates were separated via SDS-PAGE (NuPAGE Novex 4-to-12% bis-Tris gels; Thermo Fisher Scientific) and imaged on a Typhoon gel imager (GE Healthcare). Gels were stained with colloidal blue (Life Technologies, Inc.) or Instant-Blue (Expedeon) Coomassie stains to verify equal protein loading.

For all enrichments, cysteines were reduced by addition of 10 mM dithiothreitol (DTT) for 20 min at room temperature, and free thiols were alkylated by addition of 100 mM chloroacetamide for 30 min in the dark. For the comparison between *P*_*rpoS*_:*nll-mc* and *P*_*trc*_:*nll-mc* biofilms, 0.5 mg of protein lysate per sample was treated with 12 μM DBCO–sulfo-biotin (Click Chemistry Tools) in 0.5 ml PBS (137 mM NaCl, 2.7 mM KCl, 10 mM Na_2_HPO_4_, 1.8 mM KH_2_PO_4_) for 15 min at room temperature. Proteins were precipitated with acetone at −20°C and resuspended in PBS, 0.3% SDS. A small sample (~20 µg) of each treated lysate was separated by SDS-PAGE, transferred to nitrocellulose, and probed for biotin conjugation with streptavidin-Alexa Fluor 488 (Thermo Fisher Scientific). The streptavidin UltraLink resin (Pierce Biotechnology) was washed twice with PBS and added to biotinylated lysates, and this mixture was incubated overnight at 4°C. Resin was transferred to microcentrifuge spin columns (Pierce Biotechnology) and washed twice with 1% SDS in PBS and once with 0.1% SDS in PBS. Proteins were eluted by incubation with 1 mM biotin at 65°C for 20 min. Eluted proteins were separated via SDS-PAGE and subjected to in-gel digestion coupled with LC-MS (GeLCMS).

For the comparison between ciprofloxacin-treated samples, lysates were reduced and alkylated as described above. For each sample, approximately 0.5 mg of protein in 0.5 ml of PBS was incubated with 50 μl of a DBCO-agarose bead 50% slurry (Click Chemistry Tools) for 2.5 h at room temperature. Beads were washed extensively in gravity flow columns (Bio-Rad) with 40 ml (8 volumes of 5 ml) each of (i) PBS, 0.8% (wt/vol) SDS; (ii) 8 M urea; and (iii) 20% (vol/vol) acetonitrile (ACN) in water. Beads were resuspended in 50 mM ammonium bicarbonate (AB) for on-bead tryptic digestion (see the LC-MS/MS section for details).

### Imaging of planktonic cells.

For imaging of planktonic cells, cell cultures were pelleted, washed in PBS once, and resuspended in PBS. Cell suspensions were placed on 4% agarose pads, covered with a coverslip, and imaged immediately.

### Imaging of flow cell biofilms.

For the flow cell biofilms, all treatments were applied via syringe. For GFP imaging, flow was stopped and live biofilms were incubated with 0.05 μM SYTO 62 (Thermo Fisher Scientific) for 30 min at 37°C. For mCherry imaging, flow was stopped and live biofilms were incubated with 0.05 µM SYTO 9 (Thermo Fisher Scientific) for 30 min at 37°C. To visualize Anl incorporation, biofilms were fixed by incubation with 3.7% formaldehyde for 30 min and permeabilized by incubation with 70% ethanol for 5 min on ice. Fixed biofilms were washed with 0.9% NaCl, incubated with 100 mM chloroacetamide in the dark for 30 min, and treated with 25 μM DBCO-TAMRA in PBS for 30 min. Biofilms were washed extensively with PBS to remove excess dye and counterstained with 0.05 μM STYO 9. Biofilms were imaged on a Leica TCS SPE confocal microscope with a 40× or 63× objective.

### LC-MS/MS.

For GeLCMS, gel lanes were cut into 8 pieces each and destained by alternating washes with 50 μl each of 50 mM AB and a 1:1 mixture of 50 mM AB–ACN. Proteins were reduced by incubation with 6.7 mM DTT in 50 μl 50 mM AB at 50°C for 30 min and alkylated by incubation with 37 mM iodoacetamide in 50 μl 50 mM AB at room temperature for 20 min. Gel pieces were washed with 50 μl each of 100 mM AB and acetonitrile. Proteins were digested first by addition of 300 ng endoproteinase LysC in 50 μl of 100 mM Tris-HCl at 37°C for 4 h and then by addition of 150 ng of trypsin for 16 h at 37°C. Peptides were extracted by sequential washing with 50 μl each of 1% formic acid–2% acetonitrile, 1:1 acetonitrile-water, and 1% formic acid in acetonitrile. Peptides were desalted using C_18_ ZipTips (EMD Millipore).

For on-bead digestion following BONCAT enrichment with DBCO-agarose, beads were incubated with 100 ng trypsin in 100 µl 9:1 50 mM AB:acetonitrile for 18 h at 37°C. Supernatant was collected and beads were washed twice with 100 µl 20% ACN to extract all peptides. Peptides were dried, passed through HiPPR spin columns (Thermo Fisher Scientific) to remove residual SDS, and desalted with C_18_ ZipTips.

Liquid chromatography-mass spectrometry experiments were carried out essentially as previously described (63). The *rpoS* versus *trc* experiments were performed on a nanoflow LC system, the EASY-nLC 1000, coupled to a hybrid linear ion trap Orbitrap classic mass spectrometer (Thermo Fisher Scientific) equipped with a nanoelectrospray ion source (Thermo Fisher Scientific) with the following modifications: for the EASY-nLC 1000 system, solvent A consisted of 97.8% H_2_O, 2% ACN, and 0.2% formic acid, and solvent B consisted of 19.8% H_2_O, 80% ACN, and 0.2% formic acid. For the LC-MS/MS experiments, digested peptides were directly loaded at a flow rate of 500 nl/min onto a 16-cm analytical high-performange LC (HPLC) column (75 μm inner diameter) packed in-house with Reprosil-Pur C_18_AQ 3-μm resin (120 Å pore size; Maisch, Ammerbuch, Germany). The column was enclosed in a column heater operating at 45°C. After a 30-min loading time, the peptides were separated in a solvent gradient at a flow rate of 350 nl/min. The gradient was as follows: 0 to 30% B (50 min) and then 100% B (10 min). The Orbitrap was operated in data-dependent acquisition mode to automatically alternate between a full scan (*m*/*z* 400 to 1,600) in the Orbitrap and a subsequent 10 collision-induced dissociation (CID) MS/MS scans (the top 15 method) in the linear ion trap. CID was performed with helium as the collision gas at a normalized collision energy of 35% and 30 ms of activation time. Ciprofloxacin experiments were performed on a hybrid ion trap-Orbitrap Elite mass spectrometer (Thermo Fisher Scientific) with the same acquisition method, except the top 20 ions were selected for fragmentation. The analytical column for this instrument was a PicoFrit column (New Objective, Woburn, MA) packed in-house with Reprosil-Pur C_18_AQ 1.9-µm resin (120 Å pore size; Maisch, Ammerbuch, Germany), and the column was heated to 60°C. The peptides were separated with a 120-min gradient (0 to 30% B in 120 min) at a flow rate of 220 nl/min.

### Proteomic data analysis.

Raw files were searched using MaxQuant (64) against the *P. aeruginosa* PA14 UniProt entries (5,886 sequences) and a contaminant database (246 sequences). Trypsin was specified as the digestion enzyme, with up to two missed cleavages. Carbamidomethylation of cysteine was set as a fixed modification, and protein N-terminal acetylation and methionine oxidation were variable modifications. Protein abundances were estimated with MaxLFQ (65), and for each experiment peptides were matched between runs. LFQ values were normalized and used to calculate abundance ratios between samples and to estimate variance using the limma package in R version 3.2.2 (66). *P* values were adjusted for false discovery by using the Benjamini-Hochberg procedure (67).

Spearman’s rank correlations were calculated between experimental replicates by using raw LFQ values. Principal-component analysis was performed on log_10_-transformed LFQ values and included only proteins that were identified in all 12 MS analyses (*n* = 291).

### Software used for our analyses.

Additional software packages that we used but that were not mentioned above included the following. Data processing and statistical analysis were performed with Python version 2.7.9 with NumPy version 1.9.2, SciPy version 0.15.1, Pandas version 0.16.1, and scikit-learn version 0.17. Data were plotted with Matplotlib version 1.5.1 (68) and Seaborn version 0.8.0. Microscopy and gel images were analyzed with ImageJ 64-bit version 2.0.0 (69). Figures were assembled in Adobe Illustrator CS5.

### Availability of data.

The mass spectrometry proteomics data have been deposited with the ProteomeXchange Consortium via the PRIDE (70) partner repository and assigned the data set identifiers PXD007622 and PXD007261.
