# Supplementary material for: Selective Proteomic Analysis of Antibiotic-Tolerant Cellular Subpopulations in Pseudomonas aeruginosa Biofilms
Source: mBio. 2017 Oct 24;8(5):e01593-17. doi: 10.1128/mBio.01593-17 (PMC5654934; doi:10.1128/mBio.01593-17)
Supplement: FIG S1 [file mbo005173553sf1.pdf]

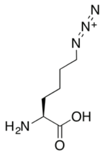

L-azidonorleucine
(Anl)

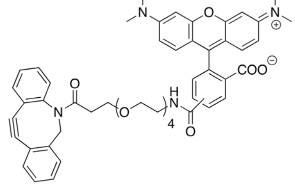

aza-dibenzocyclooctyne-
carboxytetramethylrhodamine
(DBCO-TAMRA)

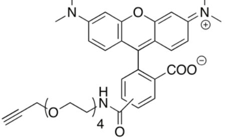

alkyne-carboxytetramethylrhodamine
(alkyne-TAMRA)

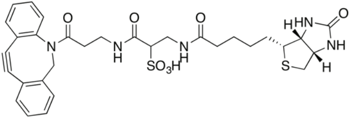

aza-dibenzocyclooctyne-sulfo-biotin
(DBCO-sulfo-biotin)
