# Supplementary figures and images for: Selective Proteomic Analysis of Antibiotic-Tolerant Cellular Subpopulations in Pseudomonas aeruginosa Biofilms
Source: mBio. 2017 Oct 24;8(5):e01593-17. doi: 10.1128/mBio.01593-17 (PMC5654934; doi:10.1128/mBio.01593-17)

A

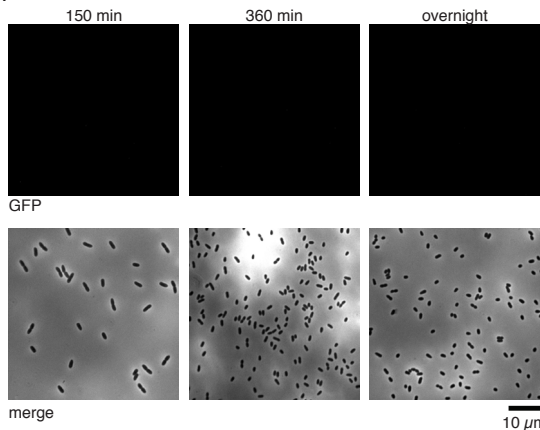

B

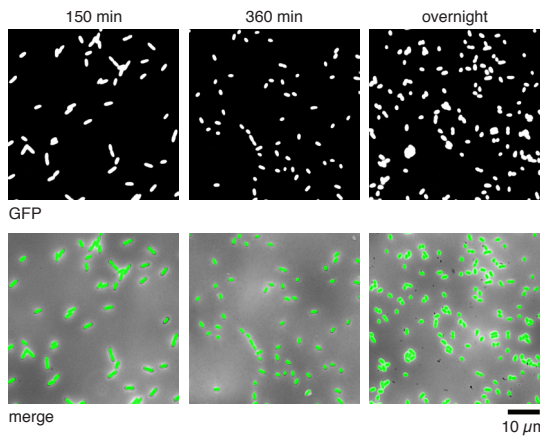

C

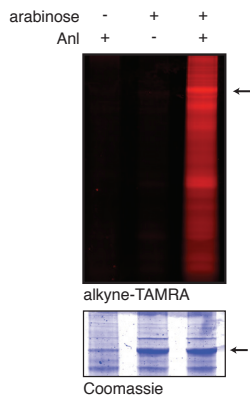

Supplement: FIG S2 [file mbo005173553sf2.pdf]

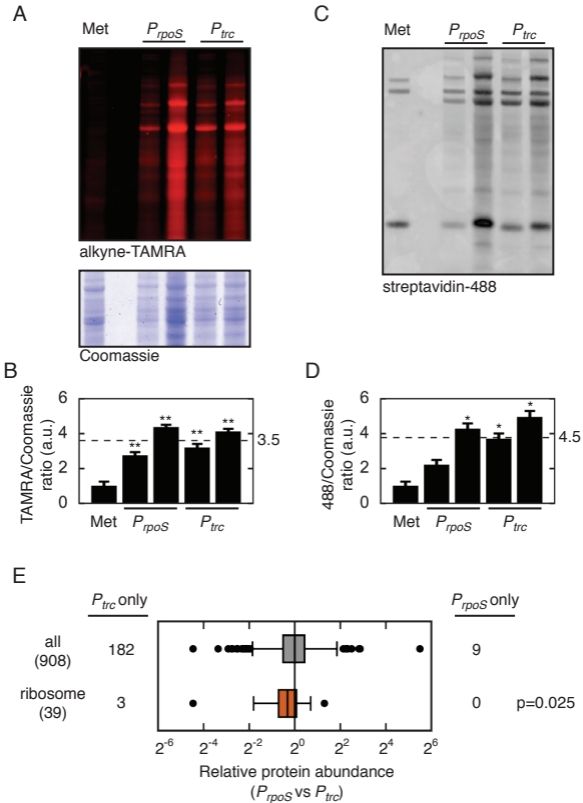

Supplement: FIG S3 [file mbo005173553sf3.pdf]

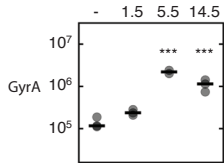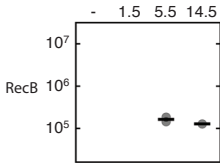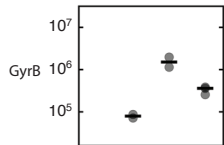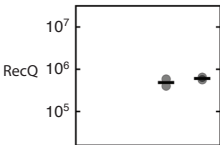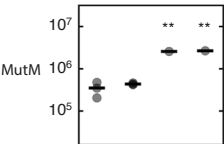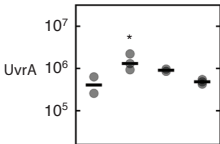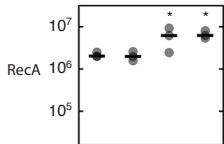

Supplement: FIG S4 [file mbo005173553sf4.pdf]
