# Supplementary material for: Selective Proteomic Analysis of Antibiotic-Tolerant Cellular Subpopulations in Pseudomonas aeruginosa Biofilms
Source: mBio. 2017 Oct 24;8(5):e01593-17. doi: 10.1128/mBio.01593-17 (PMC5654934; doi:10.1128/mBio.01593-17)
Supplement: TABLE S3 [file mbo005173553st3.docx]

| *Pseudomonas aeruginosa* strains | | |
| --- | --- | --- |
| Name | Genotype | Source |
| DKN263 | *P. aeruginosa* UCBPP-PA14 |  |
| *P_rpoS_:gfp* | UCBPP-PA14 attTn7::mini-Tn7T-*Gm^R^* *P_rpoS_:gfp* | This study |
| *Ptrc:gfp* | UCBPP-PA14 attTn7::mini-Tn7T-*Gm^R^ Ptrc:gfp* | This study |
| *P_rpoS_:nll-mc* | UCBPP-PA14 attTn7::mini-Tn7T-*Gm^R^* *P_rpoS_:nll-mc* | This study |
| *P_trc_:nll-mc* | UCBPP-PA14 attTn7::mini-Tn7T-*Gm^R^* *P_trc_:nll-mc* | This study |
| *P_algP_:gfp* | UCBPP-PA14 attTn7::mini-Tn7T-*Gm^R^ P_algP_:gfp* | This study |
| ***Escherichia coli* strains** | | |
| Name | Genotype | Source |
| DKN1298 | SM10, pTNS1 | (1) |
| DKN1299 | HB101 | (1) |
| BMB6 | Mach1 pUC18T-mini-Tn7T-*Gm^R^ P_rpoS_:gfp* | This study |
| BMB7 | Mach1 pUC18T-mini-Tn7T-*Gm^R^ Ptrc:gfp* | This study |
| BMB8 | Mach1 pUC18T-mini-Tn7T-*Gm^R^ P_rpoS_:nll-mc* | This study |
| BMB9 | Mach1 pUC18T-mini-Tn7T-*Gm^R^ P_trc_:nll-mc* | This study |
| BMB10 | Mach1 pUC18T-mini-Tn7T-*Gm^R^ P_algP_:gfp* | This study |
| BMB11 | Mach1 pUCP18 | (2) |
| BMB12 | Mach1 pBAD18-NLL-MetRS | This study |
| BMB13 | Mach1 pBADP-NLL-MetRS | This study |

1. **Choi KH, Schweizer HP.** 2006. mini-Tn7 insertion in bacteria with single attTn7 sites: example *Pseudomonas aeruginosa*. Nature Protocols **1:**153-161.

2. **West SE, Schweizer HP, Dall C, Sample AK, Runyen-Janecky LJ.** 1994. Construction of improved *Escherichia*-*Pseudomonas* shuttle vectors derived from pUC18/19 and sequence of the region required for their replication in *Pseudomonas aeruginosa*. Gene **148:**81-86.
